# Opinion: No. Ten reasons that there will be no new pharmacologic therapies for erectile dysfunctionin the foreseeable future

**DOI:** 10.1590/S1677-5538.IBJU.2015.05.03

**Published:** 2015

**Authors:** Ira D. Sharlip

**Affiliations:** University of California San Francisco School of Medicine, San Francisco, CA.

**Keywords:** Erectile Dysfunction, Therapeutics, PDE-5 Inhibitors

## BACKGROUND

From the 1960s to the 1990s, breakthrough developments in sexual medicine occurred about once every ten years. In the decade of the 1960s, there was the launch of birth control pills for women and the resulting sexual revolution. This was followed by penile implant surgery in the 1970s, intracavernous injection therapy in the 1980s and oral therapy with phosphodiesterase type 5 (PDE5) inhibitors in the late 1990s. Landmark developments since then have been the approval in 2013 of collagenase clostridium histolyticum for intralesional treatment of Peyronie's disease and the very recent approval in 2015 of flibanserin for the treatment of hypoactive sexual desire in pre-menopausal women. However, no landmark development has occurred for the treatment of erectile dysfunction since the introduction of PDE5 inhibitors starting in 1998. Moreover, there are no clinically relevant innovations on the horizon for erectile dysfunction. It is likely that current PDE5 inhibitors and intracavernous agents will be the last pharmacologic therapies for erectile dysfunction in the foreseeable future. There are ten reasons for this.

### 1. Absence of innovative therapies using new molecular targets

The first reason is that no innovative therapy using a molecular target other than PDE5 has reached any stage of clinical application. Since the molecular biology and physiology of erectile function and dysfunction have been clearly defined, there has been plenty of time for development of strategies to treat erectile dysfunction other than PDE5 inhibition, yet no new therapeutic options have emerged. Among other therapeutic targets, perhaps the most promising has been the Rho kinase system. While this system seemed to hold some potential for treatment of erectile dysfunction, it has not borne fruit for clinical development. Moreover, there are no other innovative treatment options on the clinical horizon.

### 2. Saturation of PDE5 inhibitor market

At this point, the market for PDE5 inhibitors is saturated with widely known and recognized products. Further development of PDE5 inhibitors is unlikely to occur because of this saturation. Currently there four PDE5 inhibitors which are available on many continents, two others which are available only in Asia and one other in Brazil. Two of these PDE5 inhibitors, sildenafil and tadalfil, control the great majority of the worldwide market for oral therapy of erectile dysfunction. Each of the other five products its own unique characteristics but, in reality, they do not differ greatly from the market leaders in their clinical effects. Unless a new PDE5 inhibitor can deliver distinctly advantageous characteristics, no new oral therapy for erectile dysfunction based on PDE5 inhibition will enter this saturated market.

### 3. High degree of clinical efficacy of PDE5 inhibitors

The third reason that PDE5 inhibition is likely the last oral therapy for erectile dysfunction is the clinical efficacy of PDE5 inhibitors. While currently available PDE5 inhibitors are effective for about two-thirds of men with erectile dysfunction, it is unlikely that any new PDE5 inhibitor or any other oral therapy for ED will successfully treat the one-third of men who have failed PDE5 inhibitor therapy. This is because most of the failures occur in men who have either (1) advanced fibrosis of the corpora cavernosa, for which no oral therapy can deliver sufficient PDE5 inhibitor serum levels to be effective, or (2) psychogenic problems, for which oral PDE5 inhibition or intracavernous therapy are not the therapies of choice.

### 4. High cost of drug development

The fourth reason that there will not be new drug therapy for erectile dysfunction is the huge cost in the United States to develop a new drug, which now approaches one billion US dollars. With entrenched, successful, safe and proven PDE5 inhibitors already in place and the PDE5 inhibitor market saturated, the possibility of recouping the cost of drug development is small to non-existent.

### 5. Limited size of erectile dysfunction market

The fifth factor inhibiting development of innovative therapies is the unexpectedly limited size of the market for treatment of ED. When Viagra was first launched in 1998, clinicians and pharmaceutical executives expected that all men with ED would desire treatment. Using epidemiologic evidence from the 1990s, it was estimated that there were about 30 million men in the United States alone and at least 150 million worldwide who had erectile dysfunction. It was expected that most if not all of them would want to use Viagra. While the estimates of the number of men who have erectile dysfunction are probably correct, the concept that all men with erectile dysfunction want treatment turned out not to be true. Clinical experience over the last 17 years since Viagra was launched in 1998 has shown that perhaps only one-third of men with erectile dysfunction use PDE5 inhibitors for treatment. Men who have erectile dysfunction are often curious and may try a PDE5 inhibitor once or twice but the majority of men with erectile dysfunction eschew ongoing treatment because of decreased interest in sex, absence of an interested sexual partner, contraindications due to medical co-morbidities, cost of treatment and/ or embarrassment at having to identify themselves as having erectile dysfunction in order to obtain and fill a prescription.

### 6. Limited prospects for increase in market size

A corollary to the limited size of the PDE5 inhibitor market is that there are no factors which indicate that the market size will expand in a significant way. After fifteen-plus years of availability of PDE5 inhibitor therapy, large pharmaceutical companies have had extensive experience with advertising and publicity for PDE5 inhibitors. There appear to be no new techniques which promise to increase PDE5 inhibitor sales in developed regions of the world. In developing regions, widespread poverty, the cost of treatment and the need to allocate medical assets to life-threatening conditions put market expansion out of reach.

### 7. Impending transition of Viagra to generic status

The seventh factor is the end of patent protection and the impending transition of Viagra and perhaps other PDE5 inhibtors to generic status. When this happens, it is likely that the cost of sildenafil and possibly other PDE5 inhibitors to the consumer will fall. This means that if a new drug for erectile dysfunction were to be developed, it would have to compete with the reduced cost of generic PDE5 inhibitors. This will be a powerful inhibitor of new drug development. The only way a new drug might be able to compete successfully with generic sildenafil or other generic PDE5 inhibitors is to provide a unique characteristic such as very rapid onset, much stronger effect, reduced side effects or significantly less cost compared to generic sildenafil. These problems are likely to eliminate development of a new competitor for generic PDE5 inhibitors.

### 8. Possible transition of PDE5 inhibitors to over-the-counter status

The eigth reason that a new drug for erectile dysfunction will not be developed is the possibility that one or more of the current PDE5 inhibitors will become over-the-counter products not requiring a prescription in the next few years. Lack of anonymity in purchasing prescription drugs is a very strong inhibitor of PDE5 inhibitor purchase. Many men are too embarrassed to go to a doctor and/or to their local pharmacy and be revealed as a man with erectile dysfunction. An over-the-counter PDE5 inhibitor might be sold online or can be packaged in an anonymous method for presentation at the point of purchase, eliminating the steps of a costly visit to a doctor and submission of a prescription to a pharmacy. New therapies will have to be prescription drugs while the availability of generic and/or over-the counter PDE5 inhibitors will inhibit sales of prescription drugs.

### 9. Limited market size for intracavernous injection therapy

These same arguments can be advanced for development of new intracavernous agents for treatment of erectile dysfunction. Papaverine, phentolamine and especially prostaglandin E1 are sufficiently effective that new intracavernous agents are unnecessary. The best possibilities for success of a new intracavernous agent would be a drug that would be signficantly less expensive than current products and/or a drug that would not need to be refrigerated. However, the size of the market for intracavernous drugs is small enough that there is little incentive for development of a new intracavernous agent.

### 10. Cost of compounded intracavernous injections is much less than cost of PDE5 inhibitors

An additional inhibitor to new oral drug development in the United States is the relatively low cost of intracavernous injection agents such as prostaglandin E1, phentolamine and/or papaverine that can be obtained from compounding pharmacies. Lesser expensive drugs such as these compounded agents provide another element of price competition which will discourage prospective developers of new oral or intracavernous agents for the treatment of erectile dysfunction.

## SUMMARY

In summary, the process of developing a new oral or intracavernous agent to treat erectile dysfunction is fraught with such overwhelming impediments that it is very unlikely, if not impossible, for a new drug to emerge in the foreseeable future. What is available now is the end of the line for pharmacologic treatment of erectile dysfunction.

